# Abusive head trauma: experience improves diagnosis

**DOI:** 10.1007/s00234-020-02564-z

**Published:** 2020-10-20

**Authors:** Luciana Porto, Marco Baz Bartels, Jonas Zwaschka, Se-Jong You, Christoph Polkowski, Julian Luetkens, Christoph Endler, Matthias Kieslich, Elke Hattingen

**Affiliations:** 1 Institute of Neuroradiology, Hospital of Goethe University, University Hospital Frankfurt, Schleusenweg 2 – 16, 60528 Frankfurt am Main, Germany; 2 Department of Paediatric Neurology, Hospital of Goethe University, Frankfurt am Main, Germany; 3 Institute of Neuroradiology, University Hospital Bonn, Bonn, Germany

**Keywords:** Abusive head trauma (AHT), Non-abusive head trauma (NAHT), Benign enlargement of the subarachnoid spaces (BESS), Metabolic diseases, Differential diagnosis

## Abstract

**Purpose:**

The diagnosis of abusive head trauma (AHT) is complex and neuroimaging plays a crucial role. Our goal was to determine whether non-neuroradiologists with standard neuroradiology knowledge perform as well as neuroradiologists with experience in pediatric neuroimaging in interpreting MRI in cases of presumptive AHT (pAHT).

**Methods:**

Twenty children were retrospectively evaluated. Patients had been diagnosed with pAHT (6 patients), non-abusive head trauma-NAHT (5 patients), metabolic diseases (3 patients), and benign enlargement of the subarachnoid spaces (BESS) (6 patients). The MRI was assessed *blindly*, i.e., no clinical history was given to the 3 non-neuroradiologists and 3 neuroradiologists from 2 different institutions.

**Results:**

*Blindly*, neuroradiologists demonstrated higher levels of sensitivity and positive predictive value in the diagnosis of pAHT (89%) than non-neuroradiologists (50%). Neuroradiologists chose correctly pAHT as the most probable diagnosis 16 out of 18 times; in contrast, non-neuroradiologists only chose 9 out of 18 times. In our series, the foremost important misdiagnosis for pAHT was NAHT (neuroradiologists twice and non-neuroradiologists 5 times). Only victims of motor vehicle accidents were blindly misdiagnosed as pAHT. No usual household NAHT was not misdiagnosed as pAHT. Neuroradiologists correctly ruled out pAHT in all cases of metabolic diseases and BESS.

**Conclusion:**

MRI in cases of suspected AHT should be evaluated by neuroradiologists with experience in pediatric neuroimaging. Neuroradiologists looked beyond the subdural hemorrhage (SDH) and were more precise in the assessment of pAHT and its differential diagnosis than non-neuroradiologists were. It seems that non-neuroradiologists mainly assess whether or not a pAHT is present depending on the presence or absence of SDH.

## Introduction

The published review of the literature on “abusive head trauma” (AHT) in infants by the Swedish Agency for Health Technology and Assessment of Social Services (SBU) [1] triggered an intense discussion on this issue. The main conclusion of the report was that there is insufficient scientific evidence on which to assess the diagnostic accuracy of the “triad” (encephalopathy, subdural hemorrhage (SDH), and retinal hemorrhage) in identifying traumatic shaking (very low-quality evidence). The review stressed the amount of poor-quality studies [1]. To add more confusion, in a recent paper, Debelle et al. [2] underpin the serious flaws of the SBU report.

The diagnosis of presumed AHT (pAHT) is complex and should always be the result of an extensive multidisciplinary approach. Nonetheless, neuroimaging plays a crucial role. Even though there are controversies; the main way for a radiologist to blindly assess pAHT is to evaluate the presence of the classical “triad” on magnetic resonance imaging (MRI): SDH, retinal hemorrhage (if possible), and parenchymal injuries. Enormous amounts of *clinical* experience, after an extensive multidisciplinary workup, suggests that when a young infant presents with hypoxic brain injury (HBI), SDH, and retinal hemorrhage, AHT is the most likely cause. Nevertheless, an experienced pediatric neuroradiologist will not only evaluate the triad but also other findings, such as the presence of ruptured and thrombosed cortical veins, ligamental injuries, myelination, or specifically the location and timing of the SDHs, as well as the age of the different brain injuries.

It is important for non-neuroradiologists and neuroradiologists to know that shaking is a contributor to AHT and that the old terminology “shaken baby syndrome” is a subset of AHT, i.e., AHT without impact. AHT is a well-recognized brain injury caused by the directed application of force to an infant or young child [3, 4]. Cases must be looked at individually [4]. A multidisciplinary child protection team evaluates the case and if suspicions are confirmed, the case goes to court. An independent “expert” is usually appointed to assess the case, make a report, and give “expert opinion” in court. In most countries, experts are appointed by courts of law based on their relevant experience in the field on which they will have to testify. Our premise is that increased neuroradiology experience, specifically in neuropediatrics, leads to better expertise and better reporting of complex pediatric neuroradiology imaging study in cases of pAHT. The radiologist who stands as an “expert” should be aware of the potential differential diagnoses and be familiar with the current literature and controversies. In this context, this blind study was a pilot project within two institutions to evaluate if the experience and expertise of the non-neuroradiologist and neuroradiologist could influence the quality of assessment of pAHT and its main differential diagnosis.

## Patients and methods

This retrospective study was approved by the ethics committee of Frankfurt University Hospital. “Consent” for the study was waived by the INSTITUTIONAL REVIEW BOARD/INDEPENDENT ETHICS COMMITTEE (IRB/IEC) of the Frankfurt University.

The patient data relate to a 10-year period, from January 2008 to December 2017. Cases were selected, rather than from random search, by the neuro-pediatrician who heads the task force for child abuse cases. The minimum interval between imaging and reassessment of a case in the study was more than 1 year. Thereby, we avoided that the neuroradiologists initially involved in the clinical diagnosis could remember the case and thus be influenced in their decisions. In addition, non-neuroradiologists and neuroradiologists from 2 institutions evaluated the MRI images.

Following pre-established inclusion and exclusion criteria (see Table 1) and taking into account all clinical information, including history and court results, the cases were assigned into four different groups, placed in random order and pseudo-anonymized. We excluded cases from the study which lacked clear evidence.

**Table 1:**
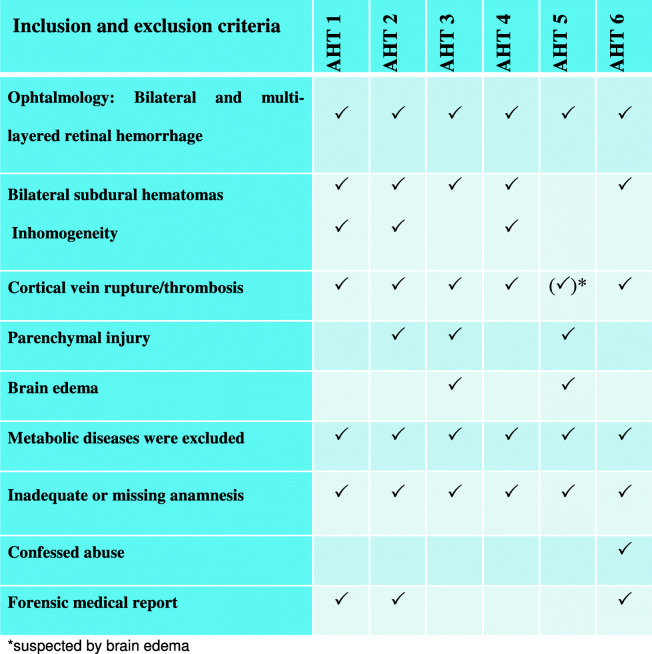
Inclusion and exclusion criteria in the abusive head trauma group

*Suspected by brain edema

Table 1 gives an overview of the criteria for AHT and lists additional criteria that support the diagnosis of AHT. In three out of six cases there was also a legal medical report which, taking all findings into account, assumes AHT. One case of confessed “shaken-impact-syndrome” was included (AHT 6)

## Patients

A total of 20 children (10 male, 10 female) were included. Patients had been diagnosed with pAHT (6 patients), non-abusive head trauma (NAHT) (5 patients), metabolic diseases (1 glutaric aciduria, type 1, 1 cobalamin defect, 1 methylmalonic aciduria), and benign enlargement of the subarachnoid spaces (BESS) (6 patients).

The general mean age was 7.7 months (newborn to 36 months) with an age-appropriate grouping and an equivalent mean age of 7.7 months in the group of pAHT. At 12.7 months, the mean age of NAHT was higher than that of pAHT, but the difference between these two groups was not significant. The mean age of the other groups was also comparable at 6.3 and 4.4 months.

### Group 1: Abusive head trauma

Cases were selected by the neuro-pediatrician who is responsible for child abuse cases. All cases were discussed in a multidisciplinary team consisting at least of a neuro-pediatrician, a neuroradiologist, a pediatric radiologist, a pediatric ophthalmologist, a metabolic, and a forensic doctor. After analyzing all clinical findings, performing the recommended procedures according to the current S3 AWMF*-Guideline of 2019 [5] and ruling out other causes than a non-accidental head trauma, the probable diagnosis of “presumed AHT” was made. All potential differential diagnoses were excluded.

The goal was to include various severe degrees of pAHT. Clinically, patients presented from no symptoms to coma requiring resuscitation. One case of pAHT with impact was also included in the study. Cases were diagnosed as pAHT according to the following criteria:

1. Infants with pAHT presented with symptoms of their injury and the clinical history were incomplete, inconsistent, or incorrect. The following presentations were observed: 3 cases of a fall from a low height (≤ 0.5 m); 1 child in good general health with associated progressive macrocephaly; 1 with hydrocephalus; and 1 who presented at the emergency room without previous trauma.
2. An obligatory inclusion criterion was the presence of retinal bleeding. These had to be diagnosed by the ophthalmologist, be present on both sides, and affect several layers of the retina. Retinal hemorrhage was a crucial criterion that was collected independently of neuroradiological imaging.
3. In addition to retinal hemorrhage and inadequate or missing clinical history, patients had *MRI* positive concomitant brain and/or ophthalmological injuries, such as SDH(s), parenchymal injuries, cerebral edema, avulsed and thrombosed cortical veins, and retinal hemorrhages.
4. Metabolic diseases were ruled out.
5. In addition, spinal, skin, and skeletal injuries were evaluated but not shown to radiologists.

To note for all cases of pAHT, we used blinded data, detailed reporting on the exclusion of differential diagnoses (see Table 1), and age-appropriate grouping. We blinded our investigators by not providing any information on the patient’s clinical situation and by standardizing the image sequences [1].

We placed particular importance on precisely defining the criteria for the pAHT group according to the current state of science and not focusing exclusively on the classical triad (SDH, retinal bleeding, and encephalopathy) [1]. We considered all cases from an intra- and inter-disciplinary perspective. Put together, the clinical and radiological signs were compatible with pAHT, although individually the findings were not specific for the diagnosis. (see Table 1).

The pAHT cases (6 patients) were sub-classified as follows: (A) pAHT without impact with bilateral SDHs (3 patients)—MRI showed avulsed/thrombosed cortical veins and possible retinal hemorrhage; (B) massive pAHT without impact with cerebral edema (2 patients)—MRI showed diffusion-weighted image (DWI) lesions, avulsed and thrombosed cortical veins (1 patient, suspected in another by brain edema), parenchymal hemorrhage, and possible retinal hemorrhage in MRI (1 patient); and (C) p*AHT* with impact (1 patient).

### Group 2: Non-abusive head trauma

The main criterion for inclusion was an intracranial hemorrhage (SDH and/or subarachnoid hemorrhage (SAH)), which, together with all other clinical abnormalities, was compatible with the details of the described accident (see Table 2).

**Table2:**
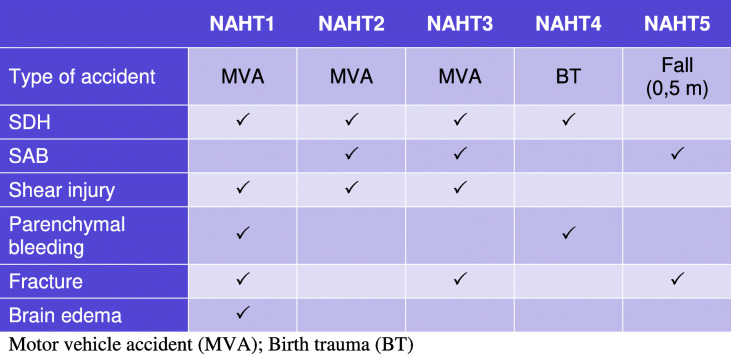
Imaging findings in non-abusive head traumas (NAHT)

Motor vehicle accident (*MVA*); Birth trauma (*BT*)

### Group 3: Metabolic diseases

The obligatory inclusion criterion was evidence of metabolic disease with the expansion of the outer cerebrospinal fluid (CSF) spaces and a disorder of myelination. One patient had glutaric aciduria, type 1; his/her MRI showed atrophy of the frontotemporal regions with enlarged subarachnoid spaces and delayed myelination. One had a cobalamin defect and one methylmalonic aciduria, both with an enlargement of the external CSF spaces and borderline delayed myelination.

### Group 4: Benign enlargement of the subarachnoid spaces

In addition to the neuroradiological evidence of dilation of the external CSF, it was important from an inclusion perspective that the patients had no neurological symptoms and that the enlargement of the subarachnoid space and the head circumference receded in the follow-up. In addition, myelination was correct for age. Six patient cases were included in this group. One of these had, in addition to the enlargement of the external CSF space, a unilateral, homogeneous, subacute subdural hematoma, which was most likely based on a minor trauma after the exclusion of coagulation disorders and metabolic disorders. There were no indications of a pAHT. The neuroradiological examination was initiated in this case to clarify macrocephaly.

## Data collection and analysis

The evaluation was performed within 2 university hospitals and included 6 examiners from 2 centers. 3 senior neuroradiologists -2 with additional pediatric neuroradiology experience, one with over 10 and the other 20 years of neuroradiology expertise; 1 pediatric neuroradiologist with over 20 years of neuroradiology expertise. 3 non-neuroradiologists with basic neuroradiology knowledge (all radiologists with completed residency in radiology and rotation in neuroradiology: one with 6 years of radiology expertise with neuroradiology rotation and in addition 1-year pediatric radiology; 2 other non-neuroradiologists with 6 years of radiology expertise with neuroradiology rotation. Important to note that all neuroradiologists had additional pediatric neuroradiology experience.

The MRI assessment was performed blindly, i.e., no clinical history was given to the radiologist. We deliberately avoided providing information about the clinical situation of the patients. To avoid bias influencing the group classification, only a small number of sequences were analyzed: fluid-attenuated inversion recovery (FLAIR) axial, T1- and T2-weighted axial images, DWI/apparent diffusion coefficient (ADC) maps, axial and coronal, and T2 sequence of a multi-echo gradient recalled echo (GRE) in the axial plane. All MRIs were performed a 1.5 T MRI. The age of the patients at the time of the examination was indicated. Specifying the age was important to assess myelination. The radiologists were not made aware of the follow-up, other imaging studies, or their results.

Evaluation of the MRI was performed in two rounds. In the first, the radiologist had to give a definitive yes/no answer to pAHT or select “pAHT could not be excluded.” In the case of a “no to pAHT” or “pAHT could not be excluded,” they should decide what other diagnoses (NAHT, metabolic disease, or BESS) they would consider. For the first evaluation round, investigators had to review the cases without the help of a questionnaire or clinical history.

For the second, detailed guidance of possible MRI findings in pAHT was included to help guide diagnosis, and the physician was then allowed to reconsider the original diagnosis. Again, clinical history was omitted.

Only the results of the first round are provided in this study, in which the investigators judged the cases alone, according to their knowledge. Our goal was to avoid the influence of specific questions (i.e., questions about retinal bleeding could guide the radiologist to the diagnosis of pAHT). In contrast, the second round was added to specifically evaluate if guidance would help.

The statistical evaluation of the collected data was carried out with the help of “Microsoft Excel” and the university’s own statistics program “Bias.” We were supported by the Institute for Biostatistics and Mathematical Modelling of Frankfurt University Hospital.

To determine the role of an MRI examination in cases of pAHT, the sensitivities and positive predictive value were determined individually for each examiner and the respective mean values for the groups of examiners. The results were then compared. The basis for the calculation was the answers from the first assessment round. To determine whether the results improved by presenting a structured answer sheet, we compared the sensitivities and positive predictive values of the first and second assessments.

We determined the consistency of the assessment within the two groups of examiners by determining Fleiss’ kappa coefficient for both groups of examiners. Here, the answers of the first assessment were relevant, in which the investigators judged the cases alone and had to determine the most probable diagnosis.

We also assessed patients individually and described specifically which cases misjudgments most commonly occurred.

## Results

One hundred twenty questionnaires were evaluated in both the first and second rounds.

Neuroradiologists showed a strong agreement in their case assessments (Graphic 1a) and more often chose correctly pAHT as the most probable diagnosis. In comparison, non-neuroradiologists were more often insecure (Graphic 1b) and frequently could not rule out AHT in the differential diagnosis.

**Graphic 1:**
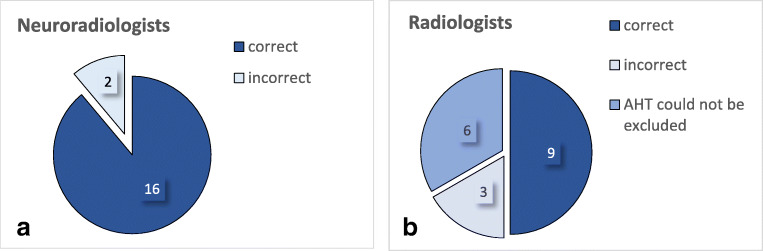
Detection of AHT by neuroradiologists (1**a**) and radiologists (1**b**) This graph only refers to patients in the AHT group (6 out of 20 patients). With 3 examiners in each group, we had a total of 18 evaluations of AHT cases for each group. Numbers shown here in dark blue correspond to the blindly correct first diagnosis of AHT. These were calculated using the first questionnaire completed by all 3 radiologists and 3 neuroradiologists.

### Group 1: Presumptive abusive head injury

The 3 neuroradiologists chose correctly pAHT as the most probable diagnosis in 16 out of 18 times. One case of pAHT with impact (AHT6, see Table 1) was misdiagnosed once as NAHT (see Fig. 3). And, one case with massive cerebral edema, but no signs of depressed skull fracture or SDH, was classified once as undefined by one neuroradiologist.

In comparison, non-neuroradiologists only detected pAHT in 9 out of 18 times (50%). Results deteriorated even further after detailed guidance of possible MRI findings was provided. Presumed AHT was assessed as NAHT seven times and twice as a metabolic disease.

Overall, neuroradiologists were blindly able to determine whether a pAHT was present or if it could be excluded (Graphics 1 and 2) more often than non-neuroradiologists. Compared with neuroradiologists, non-neuroradiologists were more often unable to exclude the possibility of pAHT.

**Graphic 2:**
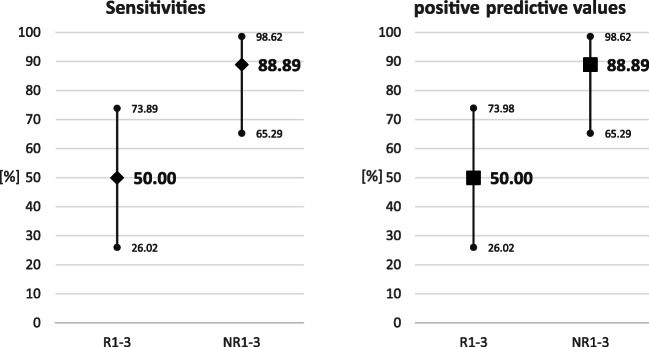
Sensitivities and positive predictive values were calculated and compared within the 2 groups: AHT-Diagnosis by neuroradiologists and radiologis Diagnostic accuracy was evaluated with sensitivities and positive predictive values. Sensitivity and positive predictive value in the diagnosis of AHT for radiologists (R1-3) and neuroradiologists (NR1-3) are shown.

The sensitivity in the diagnosis of pAHT, i.e., correctly choosing pAHT as the most probable diagnosis, was 89% for neuroradiologists and 50% for non-neuroradiologists. Results for positive predictive value, i.e., the probability of patients who have a positive MRI result actually fitting the hypothesis pAHT, were identical to the results for sensitivity.

#### Group 1A: Presumptive AHT with bilateral SDH, without impact

It was more difficult for non-neuroradiologists to diagnosis pAHT in children who presented with SDH but no parenchymal injury. In comparison, neuroradiologists reliably chose correctly pAHT as the most probable diagnosis in patients with SDH but without parenchymal injury; they correctly recognized the retinal bleeding and detected bridging vein injury associated with the SDH. None of the non-neuroradiologists recognized or suspected retinal bleeding.

#### Group 1B: Presumptive AHT with brain edema, without impact

In two cases, pAHT presented with brain edema. These cases were more often misdiagnosed. One case (AHT3, Fig. 2) with hypoxic brain edema and small compressed SDH on both sides was diagnosed as NAHT 2 out of 6 times (both times by non-neuroradiologists). One neuroradiologist correctly suspected the presence of retinal bleeding on MRI but made no group assignment. Two neuroradiologists recognized an injury of the bridge veins and assumed pAHT. Another case (AHT5) presented with hypoxic brain edema, massive shearing injuries, and no SDH. Presumed AHT was ruled out by all non-neuroradiologists but, in contrast, was recognized by all neuroradiologists. Only one non-neuroradiologist correctly suspected the presence of retinal bleeding on MRI.

#### Group 1C: Presumptive AHT with impact

One patient (AHT6, Fig. 3) had, in addition to shaking, head impact against a hard surface. Other possibility would be a hard object impact against the head. This case was very difficult to evaluate blindly. The patient presented with SDH, retinal bleeding, and rupture of the bridging veins (see Fig. 3). One neuroradiologist misdiagnosed this case as an NAHT. There was suspicion of a fracture and a soft tissue swelling (only minimal dislocation of the fracture making it difficult the evaluation on MRI, Fig. 3c). Two non-neuroradiologists misdiagnosed the case as an NAHT but did not rule out pAHT. Both investigators recognized evidence of a fracture in the MRI. What was remarkable was that none of the investigators who chose correctly pAHT as the most probable diagnosis detected the skull fracture (Fig. 3c).

### Group 2: Non-abusive head trauma

In this group, the neuroradiologists ruled out pAHT correctly in 10 out of 15 evaluations (Graphic 3). In 3 evaluations, pAHT could not be ruled out. In 2 evaluations, pAHT was wrongly assumed to be the most probable diagnosis, namely, cases “NAHT1” and “NAHT3” (see Table 2), both motor vehicle accidents (MVA).

**Graphic 3:**
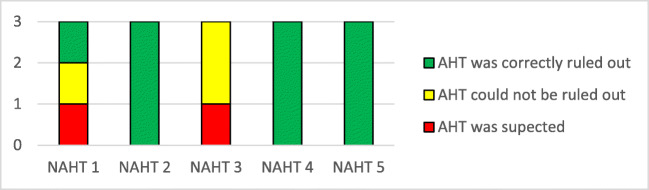
Non-abusive head trauma. Differential diagnosis by neuroradiologists

In contrast, non-neuroradiologists wrongly assumed pAHT to be the most probable diagnosis in 5 out of 15 evaluations of patients with NAHT (Graphic 4). They correctly ruled out pAHT in 6 out of 15 evaluations. And, in 4 evaluations, pAHT could not be ruled out.

**Graphic 4:**
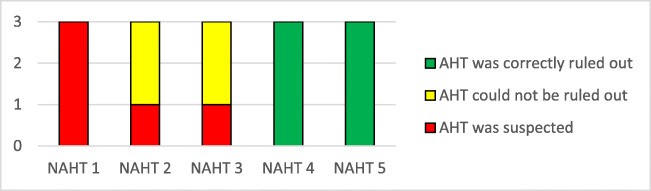
Non-abusive head trauma. Differential diagnosis by radiologists

In almost all cases where SDHs were present, the non-neuroradiologists assumed (or at least could not rule out) pAHT.

In 3 patients (NAHT1, NAHT2, and NAHT3; Table 2) with MVA, the differential diagnosis with pAHT was, blindly, particularly difficult. In these cases, pAHT was thought to be the right diagnosis 7 times and it could not be excluded 7 times (Graphics 3 and 4).

These were all victims of traffic accidents, where shear injuries were found in combination with subdural hematomas. In cases NAHT1 and NAHT3, SDHs were bilateral; in NAHT2 unilateral. In NAHT3, an injury to the bridge veins was also identified (Fig. 5). In NAHT2 (Fig. 4), all neuroradiologists were able to rule out pAHT, but none of the non-neuroradiologists did the same.

In one patient with birth trauma (NAHT4) and one who suffered a fall from a low height (NAHT5), pAHT was ruled out by all investigators.

### Group 3: Metabolic disease

In all cases of metabolic diseases, the 3 neuroradiologists were able to correctly rule out pAHT. The non-neuroradiologists correctly ruled out pAHT in 8 out of 9 case assessments. In 1 case (glutaric aciduria, type I), pAHT was wrongly assumed to be the most probable diagnosis. All other investigators were able to assign this case to a metabolic genesis.

All neuroradiologists assessed myelination as delayed in patients with metabolic diseases. Remarkably, the 2 non-neuroradiologists who correctly suspected metabolic disease did not detect any disturbance of myelination.

### Group 4: Benign enlargement of the subarachnoid spaces

Neuroradiologists correctly ruled out pAHT in all cases of BESS. Non-neuroradiologists ruled out pAHT correctly in 14 out of 18 evaluations. In 3 case assessments, pAHT was wrongly assumed to be the most probable diagnosis and in 1 evaluation pAHT could not be ruled out. One case with a widening of the external cerebrospinal fluid spaces and associated unilateral SDH proved to be, as expected, difficult for non-neuroradiologists (Fig. 6).

## Discussion

Legal cases of AHT have become more complex during the last 20 years [6]. Guidelines of AHT are usually based on evidence and consensus; the later sometimes leading to misinterpretations in court. Yet, good recommendations and reviews from expert groups [4, 7, 8] have been published and evidence behind AHT is strong. Thus, the “expert” who is appointed to assess the case and give “expert opinion” in court should be familiar with these recommendations.

The radiologist who analyzes the brain MRI performed after pAHT plays a key role in its diagnosis and differential diagnosis. Neuroradiologists, who are routinely exposed to pediatric cases, have learned after years of training to analyze the pediatric brain MR images systematically and to interpret all findings. This perception is different in a non-neuroradiologist without advanced neuroradiology training.

Our series showed that, blindly, it was only possible to distinguish pAHT from other differential diagnoses with high confidence (sensitivity of 88.89%) if the assessment of brain MRI images was carried out by a neuroradiologist with experience in neuropediatrics. This result is of particular interest in cases of unwitnessed abuse, where the brain MRI can provide crucial information with which to initiate further diagnostics. In this study, we deliberately avoided providing information about the clinical situation of the patients to the evaluator.

The level of experience of radiologists plays an especially important role when pAHT does not present itself with bilateral SDH, which is the most frequent intracranial lesion in patients with AHT [9]. Non-neuroradiologists with no experience in neuropediatrics assessed whether or not a pAHT was present depending on “the occurrence of the SDH.” This thesis is supported by the fact that in almost all cases with SDHs, the non-neuroradiologists either assumed or could not exclude the possibility of a pAHT. If a NAHT with SDH and shearing injuries was shown, none of the non-neuroradiologists could rule out a pAHT. In addition, one case of a BESS and unilateral SDH (Fig. 6; case “BESS6”) was interpreted as pAHT by two of the three non- neuroradiologists. Indeed, SDHs are observed in up to 90% of infants with AHT [9, 10], often parafalcine, and are frequently enough to raise suspicion of abuse. Although SDH is far more common after AHT, they also often occur following NAHT. The main difference is that inhomogeneous SDHs are more frequently found in AHT than in NAHT [9]. It should be emphasized that no single injury is pathognomonic for AHT, not even SDHs, and it is therefore important to consider other imaging features and history.

When compared with non-neuroradiologists, neuroradiologists based their decisions on different imaging aspects and not only on the presence or absence of SDH. They were therefore able to differentiate the cases more precisely. Even if SDHs were present, a pAHT could be excluded.

The presence of parenchymal brain injury in patients with AHT is the most significant cause of morbidity and mortality [4]. Contusions and shear injuries are more prevalent in NAHT, but lacerations and parenchymal clefs are more often seen in AHT. Hypoxic-ischemic injury is more common in AHT or significant NAHT [4]. Hypoxic-ischemic encephalopathy belongs to the classical “triad” on MRI, which probably explains why non-neuroradiologists had difficulty in diagnosing pAHT with SDH in the absence of parenchymal injury. In comparison, neuroradiologists reliably diagnosed pAHT with SDH without parenchymal injury; they correctly recognized the retinal bleeding and detected bridging vein injury associated with the SDH. In these cases, none of the non-neuroradiologists recognized or suspected the retinal bleeding.

Retinal bleeding is classically associated with AHT, but also it has other causes, such as accidental trauma. In cases of NAHT, the retina shows fewer bleeding (except in cases of severe trauma), which is usually limited to the posterior pole [4]. It is extremely rare after falls from low heights (< 3%), other than in cases of associated epidural hemorrhage or occipital impact. The incidence of retinal bleeding after vaginal birth is for example up to 37.3% [4]. This is important when evaluating the brain MRI of small children. SDH-associated retinal hemorrhages may result from accidental trauma, such as after birth.

In our data, severe NAHT was more likely to be mistaken for pAHT. It is important to emphasize that only the cases of severe MVA with bilateral SDHs (Fig. 5, case “NAHT3”) and additional shearing injuries led neuroradiologists to erroneously assume a pAHT. It is without a doubt that these cases would not even have been considered as pAHT if the previous history had been known.

It is important to note that all investigators, non-neuroradiologists and neuroradiologists, were able to distinguish between pAHT and the usual household fall, namely a fall from a low height (≤ 0.5 meters), which is the common misleading history provided by caregivers.

Neuroradiologists blindly detected 16 out of 18 evaluations of pAHT, and all cases of pAHT were correctly detected blindly by at least 2 of the 3 neuroradiologists. Furthermore, the neuroradiologists also succeeded in identifying cases of pAHT in which no or only minor SDHs were present. They were able to detect retinal bleeding more precisely in the MRI and interpret it correctly.

In contrast, non-neuroradiologists chose correctly pAHT as the most probable diagnosis only in 50% of the pAHT case assessments correctly (9 out of 18 evaluations). In 7 evaluations, pAHT was interpreted as NAHT and twice as a metabolic disease. The second round, where we tried to improve the results with specific questions such as asking about retinal bleeding, resulted in even worse results for the non-neuroradiologist group. This could be explained by the fact that compared with neuroradiologists, non-neuroradiologists are rarely exposed to such specific images, and combining specific findings into a coherent, organized way of diagnosing pAHT, is difficult.

In cases of pAHT, neuroradiologists more often suspected or detected retinal bleeding. This shows that a higher degree of experience is required to detect retinal bleeding in MRI. Teixeira et al. [11] showed the sensitivity of a SWI sequence to be estimated to be up to 80% for the detection of retinal bleeding in MRI. In relation to our study, we refrained from adding the SWI images to avoid bias, which would have indicated the presence of a traumatic genesis.

Cerebral edema can result from abusive and non-abusive trauma or from other nontraumatic causes such as meningitis or encephalitis (infections were not included). Specifically, due to the limited cerebral autoregulation, AHT is often associated with hypoxia and brain edema (Fig. 2, case “AHT3”) [12–14]. The association of AHT with brain edema and head impact makes the diagnosis of AHT more complex. These cases are extremely difficult. One case with massive cerebral edema, but no signs of depressed skull fracture, was classified as undefined by one neuroradiologist (Fig. 2; case “AHT3”). AHT is common in infants and young children with massive brain edema, which compresses the SDH in the acute phase. As a consequence, the absence of SDH in an infant or young child with massive brain edema is not enough to rule out AHT. In such cases, the clinical history is extremely helpful and follow-up MR images after swelling has receded are important. Whenever an abuse is suspected, sequential MRIs should be performed because brain injuries evolve over time.

Hypoxic brain injury, causing SDH and retinal bleeding, is one of the alternative theories proposed to explain AHT [4]. This theory has been contested by different studies [15–18] in which SDH was not observed in brain MRI or pathology in patients after hypoxia. Our patient “AHT3” (Fig. 2) showed hypoxia and massive cerebral edema associated with SDH and multiple ruptured cortical veins, after falling from a low height (≤ 0.5 m), and being resuscitated. Basically, a fall from a low height is not a reasonable explanation for hypoxia; moreover, small accidental injuries do not usually cause any neurological dysfunction. In addition, the raised intrathoracic pressure caused by resuscitation would not have been enough to cause increased intracranial and retinal venous pressure by blocking venous return, according to computer models [4, 18].

### Diagnosis to exclude

#### Accidental trauma

In our series, the foremost important misdiagnosis for pAHT was NAHT (1 out of 18 times by neuroradiologists; 7 out of 18 times by non-neuroradiologists). This is in agreement with the previous literature [4, 19–25]. It is important to note that there is a similarity of the MRI findings between these two injuries. Not only intracranial injuries but also skull fractures are commonly seen with both entities. In fact, Choudhary et al. [4], in a review, point out that skull fractures are equally common following NAHT as after AHT, but complex skull fractures are more common after AHT [4, 19–25].

In our sample, as to be expected under blind conditions, the correct recognition of a brain fracture misled the diagnosis. One patient (AHT6, Fig. 3) with pAHT and impact was misdiagnosed as NAHT: once by a neuroradiologist and twice by non-neuroradiologists. Radiologists must be aware that AHT can occur upon impact. In this scenario, infants and children under 2 years of age victims of AHT may show complex skull fractures.

In 3 patients with traffic accidents and NAHT, the differential diagnosis with pAHT was, blindly, particularly difficult. In all three cases of MVA, parenchymal injuries were found in combination with SDHs. In addition, 1 patient showed bridge vein injuries. In these patients, pAHT could have been easily ruled out if the neuro- and non-neuro-radiologist had access to clinical history.

The main problem usually faced by the neuro- and non-neuro-radiologist is the differential diagnosis with small accidental injuries at home, such as fall from a height of 0.5 m (case “NAHT5”). In these cases, the injuries and MRI findings are proportional to the height. Couches, for example, have been blamed for injuries. The reality is that children almost never die in falls from low heights and they rarely even suffer serious injuries. The specific case of a fall from a height of 0.5 m (case “NAHT5”) was identified as NAHT by all examiners, who recognized the presence of soft tissue swelling and assigned the case as accidental injury.

In an extensive review of the literature, Choudhary et al. [4] showed that severe brain injury caused by small accidental injuries at home is rare and children usually do not show any neurological dysfunction. In one study of low falls [26], there was a mortality rate of 0.48 per million per year in children younger than 5 years [4]. Significant parenchymal bleeding, contusions, and HBI are uncommon after low falls [4].

Another important differential diagnosis is birth traumatic injury (“NAHT4”). In this case, all neuroradiologists and non-neuroradiologists were able to rule out the possibility of a pAHT and correctly assumed an accidental genesis. Hemorrhage can occur after vaginal delivery or cesarean and presents as an asymptomatic thin subdural or intradural collection (< 3 mm) located posteriorly. It resolves within a month to 6 weeks and does not appear to rebleed [27]. The SDH may be symptomatic in more traumatic deliveries [27].

It is important to emphasize the radiological distinction between pAHT and NAHT. The accidental trauma presents with extra-axial hemorrhages close to the site of injury with soft tissue swelling. An epidural hematoma happens more often in cases of NAHT, but it does not rule out pAHT; in children, it is often caused by a direct traumatic violent effect. In contrast to adults, epidural hemorrhage in children is predominantly of venous origin, mostly due to injury of the sinus or the diploic veins in children with skull fractures [13, 28]. In NAHT, the SDH is normally localized focally and results from the application of greater force. A more significant accidental injury, such as MVA, can cause an acute SDH, which may be inhomogeneous due to the rapid thrombus formation within the subdural hemorrhage in association with acute bleeding or serum [12]. Nevertheless, the presence of bilateral SDHs with additional shearing injuries led some of the evaluators to assume a pAHT.

**Fig. 1:**
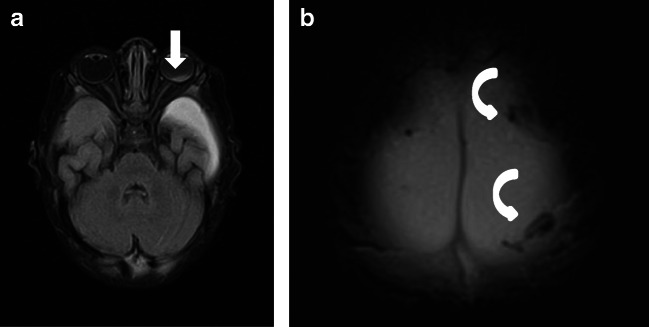
Case “AHT4”. 5 month-old. 1A: FLAIR ax, 1B:T2* ax. • Anamnesis (not available for the neuro/radiologists): Rule out hydrocephalus by macrocephaly. • MRI findings: Bilateral inhomogeneous SDH, rupture of the cortical veins (curved arrows) and retinal bleeding (straight arrow). No parenchymal injury. Extra-axial blood clots with a tubular shape in the high convexity (curved arrows) are suggestive of an acute bridging vein thrombosis and are known as “lollipop-” and “tadpole- sign” (bridging veins that terminate abruptly). • All experienced neuroradiologists correctly assumed a pAHT. Two neuroradiologists recognized the retinal hemorrhage, another neuroradiologist suspected it. • 2 out of 3 non-neuroradiologists correctly assumed an pAHT. No non-neuroradiologist recognized or suspected the retinal hemorrhage.

**Fig. 2:**
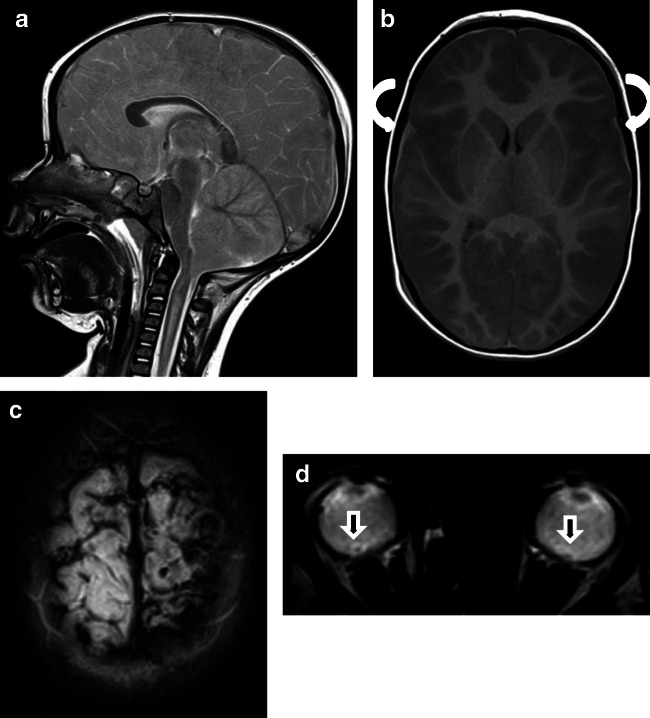
Case “AHT3”. 2 year-old. 2A: T2 weighted(w) sag, 2B:T1w ax, 2C: T2* and 2D: T2* • Anamnesis (not available for the neuro/radiologists): Small fall (≤ 0.5 meters) followed by resuscitation. • MRI findings: Massive brain edema with herniation (2A). Small bilateral SDH (curved arrows). Multiple ruptured cortical veins (2C) and suspected retinal bleeding (2D, straight arrows) • All investigators recognized bilateral subdural hematomas. • Two neuroradiologists recognized bridging vein injury, both presumed AHT, “shaking trauma”. One neuroradiologist did not recognize this case as pAHT; but made no further group assignment. • One non-neuroradiologist, who detected a retinal hemorrhage, diagnosed pAHT. However, he described no rupture of the bridging veins or injuries of the parenchyma. Using the second appraisal sheet, another non-neuroradiologist was able to detect pAHT. However, no retinal hemorrhage was described by him /her.

**Fig. 3:**
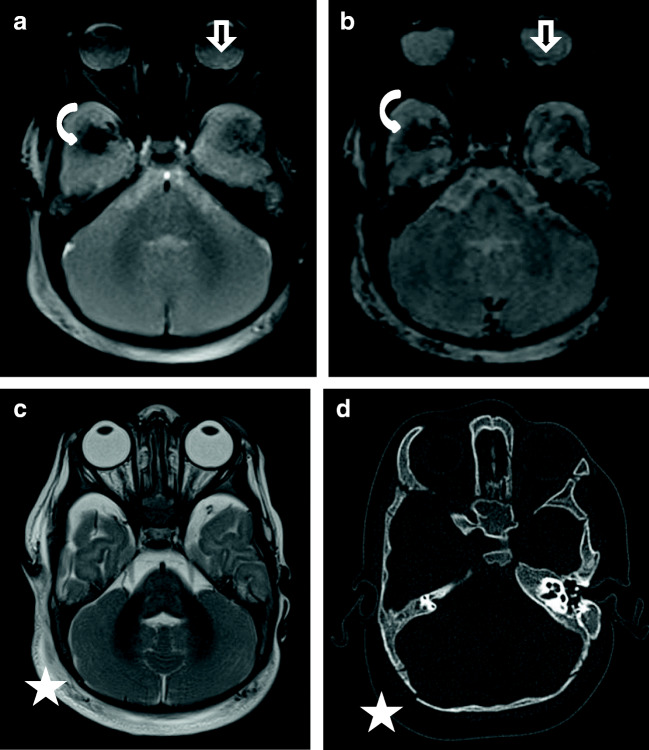
Case “AHT6”. 7 month-old (AHT 6). Presumed AHT with impact (confessed “shaken-impact-syndrome”). 3A: T2* ax, 3B: SWI ax, 3C: T2w ax and 3D: cCT, bone window. Images 3B and 3D were not available to the evaluators. SWI (3B) has a higher sensitivity for the detection of bleeding in MRI. The cortical vein rupture (curved arrow) was better delineated in SWI (3B) compared to T2* (3A). 3A and 3B show a small retinal bleeding (straight arrows). The fracture was difficult to diagnosis in MRI (see T2w:3C) compared to CT (3D). • Anamnesis (not available for the neuro/radiologists): 40 cm high fall on laminate • Due to the additional MRI findings resulting from the impact (fracture and soft tissue swelling) this case was evaluated by one neuroradiologist as an accidental injury. • Two non-neuroradiologists misdiagnosed this case as a NAHT, but did not rule out pAHT. Both investigators recognized evidence of a fracture in the MRI.

**Fig. 4:**
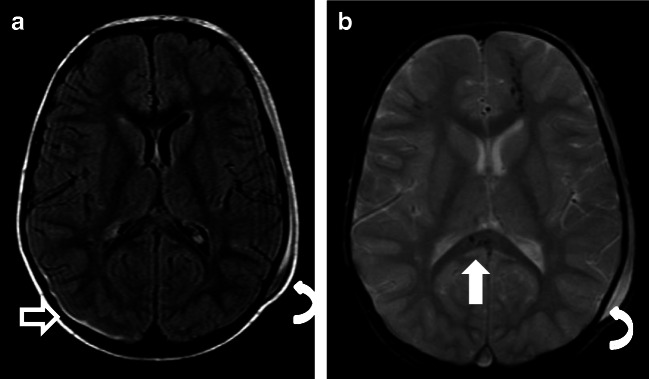
Case “NAHT2.” 2 years old. **a** FLAIR. **b** T2*. Anamnesis (not available for the neuro/radiologists): hit by a car as a pedestrian the day before. MRI findings: a coup injury with soft tissue edema on the left side (**a** and **b**, curved arrow) with a contrecoup on the opposite side due to abrupt deceleration of the head. The rapidly changing velocities within the skull may stretch and tear small bridging veins. Much more common than epidural hemorrhages, subdural hemorrhages (**a**, black straight arrow) generally result from shearing injuries due to various rotational or linear forces. Note on T2* the diffuse axonal injury (**b**, white straight arrow) with a characteristic distribution: typically located in the corpus callosum, white matter, and gray-white matter junction on the left frontal lobe. All neuroradiologists were able to rule out pAHT but none of the non-neuroradiologists

In contrast, SDH in children with AHT is usually multifocal, typically along the posterior interhemispheric fissure, close to the vertex, due to the rupture of the bridging veins (Fig. 1b, case “AHT4”; Fig. 2c, case “AHT3”) and in the posterior skull fossa [13, 28]. The inhomogeneous presentation of SDH is more common in AHT (91%) than in accidental trauma (53%) [21]. Yet, an inhomogeneous presentation of SDH (hematohygroma) does not prove the existence of multiple events but suggests a traumatic connection between the subarachnoid space and the subdural space, which is relatively common in children with AHT [29]. In order to distinguish multiple events that indicate an AHT, the detection of SDHs of different ages (different instances of trauma) in at least two different sites is vital [30]. It is important to know that the resorption of SDH varies strongly over time [10]. Epidural hematomas are rare in children with AHT but can occur (Figs. 3 and 4).

Subarachnoid, intraparenchymal, and intraventricular hemorrhage are non-specific and occur equally in AHT and NAHT [4, 22–25].

**Fig. 5:**
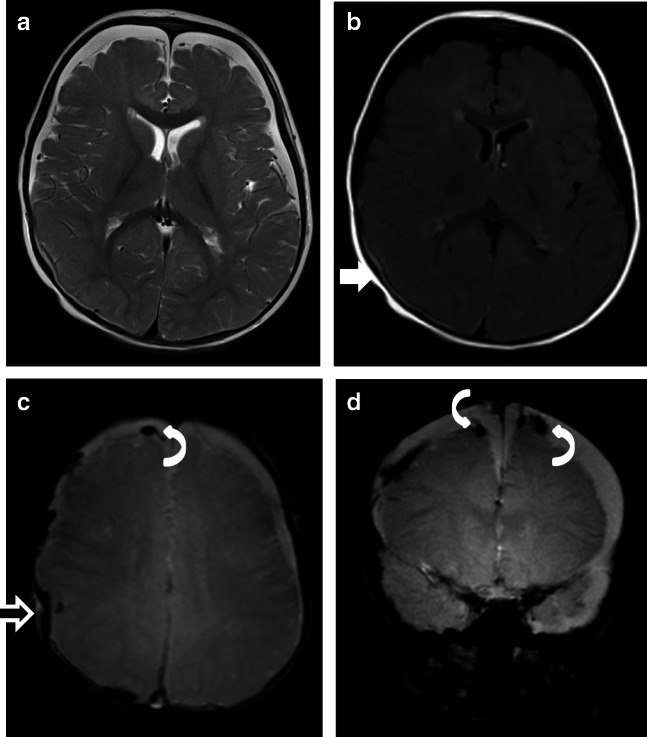
Case “NAHT3.” 1 year old. **a** T2w. **b** FLAIR. **c** T2*. **d** T2^*^ (not available for the neuro and non-neuroradiologists). Anamnesis (not available for the neuro/radiologists): MVA. Toddler on the mother’s lap and not buckled in. MRI findings: acute SDH. Axial T2 shows bilateral SDHs along the hemispheres (**a**), with the sedimentation of the blood in the dependent portion of the hematoma (**b**, white straight arrow) indicating that the SDH is from a more recent injury. In addition, the child had SAH, impression fracture of the calvarium (**c**, black straight arrow), and multiple avulsed and thrombosed cortical veins (**c** and **d**). SDH with an interhemispheric high convexity or posterior fossa location and associated thrombosed cortical veins are highly associated with pAHT. In addition, the association with impression fracture made it very difficult to differentiate NAHT from pAHT with impact. The mechanical forces during shaking and a fast acceleration-deceleration injury (toddler in mother’s lap during a car accident) are similar and might explain the MRI mimics. This case emphasizes that the presence of ruptured bridging veins is NOT pathognomonic for pAHT and can happen in cases of severe NAHT. Both groups were very uncertain in this case; 2 neuroradiologists and 2 non-neuroradiologists were not able to rule out pAHT; 1 neuroradiologist and 1 non-neuroradiologist assumed pAHT to be the correct diagnosis

The presence of ruptured bridging veins is not pathognomonic for AHT, but its presence supports a traumatic cause for the SDH [31]. Typically, disruption of the bridging veins occurs at their insertion into the sagittal sinus leading to subdural bleeding. Therefore, venous injury/thrombosis occurs not only after AHT but also following NAHT. In our sample, as to be expected, not only patients with pAHT but also those with NAHT showed ruptured cortical veins (Fig. 5, case “NAHT3”). However, the rupture of bridging veins resulting from acceleration and deceleration forces is strongly associated with AHT. In fact, up to 70% of children with AHT have some sort of venous abnormalities [4].

#### Metabolic disease

The second most common misdiagnosis within the pAHT group was a metabolic disease (2 out 18 case assessments of pAHT in the first round by non-neuroradiologists but none by neuroradiologists).

Our raters had no access to clinical information or to the head circumference, both of which play an important role in the differential diagnosis [32]. An acute increase in the head circumference speaks for AHT, whereas a constant macrocephaly with developmental delay for metabolic disease.

The neuroradiologists paid attention to the myelination stage, while none of the non-neuroradiologists did. In addition, the neuroradiologists were aware that infants with metabolic disease, such as glutaric aciduria type I (GA1), are predisposed to develop SDH. The relatively high estimated incidence of SDH (20–30%) [33] in children with GA1 is probably due to frontal lobe atrophy with stretching of cortical veins and also to neurotoxic products.

#### Benign enlargement of the subarachnoid spaces

The evaluation of normal myelination is important. In this pilot project, no metabolic disease or BESS was misdiagnosed as pAHT by neuroradiologists. BESS is a common finding in imaging studies indicated by macrocephaly in infancy and the recognition thereof is important. No misdiagnosis was made by neuroradiologists. In comparison, non-neuroradiologists had difficulties in correctly ruling out pAHT in cases of BESS, especially when it was associated with SDH.

**Fig. 6:**
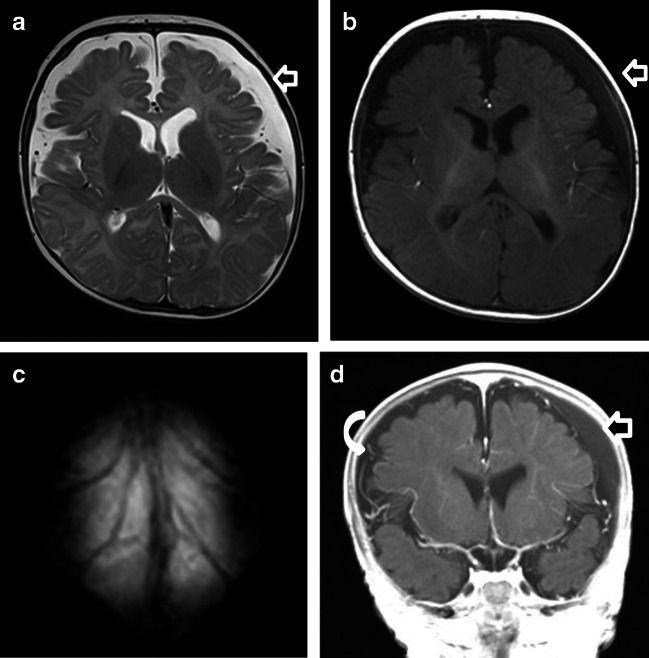
Case “BESS6.” 4 months old. **a** T2-w ax. **b** T1-w ax. **c** T2* ax. **d** T1-w after contrast (gadolinium was given by wish of the pediatrician). Anamnesis (not available for the neuro and non-neuroradiologists): macrocephaly. MRI findings: on the left, the T1-w hyperintense SDH (**b** and **d**) displaces the leptomeningeal vessels inward and away from the inner table. In comparison, BESS (curved white arrows) on the right (**d**) demonstrates interdigitating vasculature within them and is iso-attenuating to the cerebrospinal fluid (CSF) without mass effect. In this patient, the association between BESS and homogenous late subacute SDH left frontoparietal (**a**, **b**, and **d**; straight arrow) led twice to the wrong assumption of an pAHT within the group of non-neuroradiologists. All neuroradiologists correctly assumed BESS and ruled out pAHT. They based their diagnosis on the absence of avulsed and thrombosed cortical veins (see T2* (**c**) and SWI (not shown)) and the normal pattern of myelination as assessed by T1-w imaging (**b**)

Differentiating between SDHs and enlargement of the subarachnoid spaces is one of the most important responsibilities when interpreting brain imaging in infants. The distinguishing feature by which BESS and SDH can be differentiated is the location of the bridge veins (Fig. 6) [9]. In patients with SDH, the bridging veins are shifted to the brain surface (Fig. 6d, case “BESS6”). In the case of BESS, there is an enlargement of the outer cerebrospinal fluid spaces. The veins run through the subarachnoid space and can be detected directly under the skull (Fig. 6d). Interestingly, in case 6, the absence of bridging vein rupture led to the diagnosis of absence of pAHT; to date, there are no studies to support this conclusion, only clinical/radiological experience.

It is questionable if BESS predisposes SDH. According to a recent review [4], only around 5% of infants with BESS develop subdural collections and only 1.7% are hemorrhagic. The task force for child abuse from the pediatric radiology society suggests that when children younger than 2 years of age with BESS present with subdural collections, further evaluation should be performed to exclude traumatic cause, including AHT (as we did with our patient).

## Conclusion

It appears that non-neuroradiologists assess whether or not a pAHT is present mostly depending on “the presence of subdural hematomas.” The presence or suspicion of retinal bleeding played a minor or no role in the evaluation of the non-neuroradiologists.

Neuroradiologists as a rule looked beyond SDH and were consequently more precise in assessing pAHT and its differential diagnosis than non-neuroradiologists. Basically, neuroradiologists with pediatric expertise make the diagnosis of pAHT not only based on the “triad” (SDH, retinal hemorrhage, and HBI) but also on other findings, such as ruptured and thrombosed cortical veins, the presence of SDHs in different locations, and age and/or parenchymal injuries with different ages. In addition, they routinely evaluate myelination. All neuroradiologists were able to rule out all cases of birth trauma, a fall from a low height, BESS, and metabolic diseases.

In cases of traffic accidents and NAHT, the differential diagnosis with pAHT was, blindly, particularly difficult, even for neuroradiologists, due to the differential diagnosis with pAHT associated with impact. But after a MVA, medical history would not give any room for such misdiagnosis.

Despite extensive literature in neuroimaging of AHT, the early presumption of AHT remains a challenge not only for non-neuroradiologists but also for neuroradiologists. The final diagnosis of presumptive AHT can never be based only on radiological findings and should never be based on a single injury. Presumptive AHT should be viewed as a combination of all imaging findings, including extracranial injuries, in the overall clinical context. Other diseases which cause similar brain MRI findings should be ruled out. In addition, the diagnosis should be made inter-disciplinary, based on medical, forensic, and social investigation.

The results of our study should be viewed critically due to the large confidence intervals. It was a pilot project and can be used as the basis for future data collection in a multicenter prospective study.
